# Multiple-Yolk–Shell NiO Microspheres for Selective Detection of *m*-Xylene

**DOI:** 10.1021/acsami.4c09428

**Published:** 2024-10-05

**Authors:** Reinaldo dos Santos Theodoro, Gustavo Sanghikian Marques dos Santos, Bruna Soares de Sá, Tarcísio Micheli Perfecto, Diogo Paschoalini Volanti

**Affiliations:** † Laboratory of Materials for Sustainability (LabMatSus), São Paulo State University (UNESP), Rua Cristóvão Colombo 2265, São José do Rio Preto 15054-000, Brazil; ‡ Brazilian Agricultural Research Corporation (EMBRAPA), São Carlos SP 13560-970, Brazil; § Brazilian Center for Research in Energy and Materials (CNPEM), Campinas, SP 13083-970, Brazil

**Keywords:** multishell structure, nickel oxide, chemoresistive, microwave synthesis, volatile organic compounds, selectivity

## Abstract

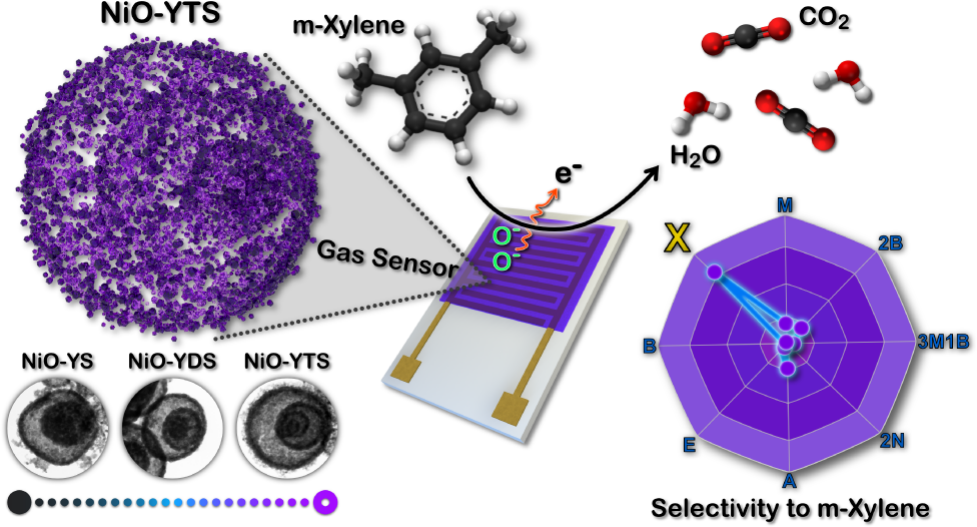

*m*-Xylene is a volatile organic compound that is extensively used in various industrial processes. It is toxic, posing significant risks to human health and the environment. Therefore, developing gas sensors with high sensitivity and selectivity for *m*-xylene detection is critical. In this work, we demonstrated the synthesis of NiO-yolk double-shell (NiO-YDS) and NiO-yolk triple-shell (NiO-YTS) derived from NiO/Ni-BTC and NiO/Ni-PTA composites, respectively, using the microwave-assisted solvothermal method from Ni-BTC-derived NiO spheres. The NiO/Ni-BTC composite has trimesic acid (H_3_BTC) as an organic linker, while NiO/Ni-PTA has *p*-terephthalic acid (PTA). We investigated the sensing properties of these materials for 2-butanone, 2-nonanone, 3-methyl-1-butanol, acetone, benzene, ethanol, methanol, and *m*-xylene. These composites exhibited excellent sensitivity and selectivity for detecting *m*-xylene under dry conditions. Specifically, the NiO-YTS sensor showed a sensitivity of 217.5% to *m*-xylene, while the NiO-YDS sensor demonstrated a sensitivity of 179.8% at 350 °C in dry air. We emphasize the NiO-YTS composite due to its superior sensitivity and selectivity in detecting *m*-xylene compared with the NiO-YDS composite. The NiO-YTS sensor exhibited stable and reproducible sensing performance for 100 ppm of *m*-xylene under optimum working conditions, with a theoretical detection limit of 5.43 ppb and relatively fast response time (89 s) and recovery time (191 s). This work describes an easy method for synthesizing NiO-YDS and NiO-YTS derived from NiO/Ni-BTC and NiO/Ni-PTA composites. It demonstrates that these composites represent a new class of materials that can potentially enhance the sensitivity and selectivity of *m*-xylene gas sensors.

## Introduction

The solvent xylene is a crucial raw material extensively utilized across various industries, particularly in synthesizing materials and producing daily items such as plastics, leather, fabrics, and paper. Xylene is also present in adhesives, cleaning agents, gasoline, and cigarettes.^1,2^ Despite its widespread use, the World Health Organization (WHO) classifies xylene as a toxic compound posing severe health risks.^3^ Exposure to xylene gas can lead to adverse effects on the respiratory system, kidneys, nervous system, heart, and lungs, potentially causing severe pulmonary problems, pulmonary cancer, leukemia, and other illnesses. Moreover, inappropriate discharge of this VOC into nature poses a significant threat to the environment.^3−5^ Therefore, developing a device that can supervise and detect xylene species in specific locations is both a challenge and a crucial necessity, which is particularly important, since detecting xylene gas with the human olfactory senses is challenging.

In recent years, semiconducting metal oxide (SMO)-based gas sensors have gained significant importance and attention. They have been extensively used to detect volatile organic compounds (VOCs) in numerous applications, including air pollution monitoring, disease diagnosis, industrial protection, food analysis, ambient protection, and more.^6−8^ SMO gas sensors are highly promising due to their low cost, accessibility, compact design, synthetic ease, and low-energy consumption. These sensors can detect many VOCs, such as xylene, more quickly and in real time than conventional techniques, such as gas chromatography and liquid chromatography coupled to mass spectrometry (GC-MS and LC-MS).^9,10^ Despite their excellent capability, gas sensors based on SMO have some limitations, mainly due to their difficulty in detecting gases with sensitivity, selectivity, and stability in different environments. New strategies for improving sensor performance include employing noble metals as catalytic filters, producing mixed-phase materials in heterostructures, building distinctive morphologies using metal–organic frameworks (MOFs) as precursors, and utilizing MOFs as filters on pure metal oxides (SMO@MOF composites).^11−13^ The latter represents a rare example where organic–inorganic hybrid layers have been reported to improve gas response and selectivity.^14^

Numerous studies have explored NiO-based gas sensors for detecting xylene gas. However, these studies focus on gas sensors derived from Ni-MOFs, heterostructures, or noble metals as catalytic filters.^15,16^ Despite the numerous studies on NiO-based sensors for detecting xylene and other gases, none of them have delved into the exploration of SMO@MOF composites with NiO, such as NiO/Ni-metal–organic-framework (NiO/Ni-MOF), for use as a gas sensor. As mentioned, this material class is seldom employed in gas sensors with only a handful of studies investigating ZnO@ZIF-8 as a gas sensor.^17,18^ Although new studies have been conducted on these SMO@MOF composites for use as gas sensors due to their recent evaluation, this class of materials shows excellent potential in enhancing sensing performance, particularly in improving sensitivity and selectivity standards. Just like ZIF (2-methylimidazole) mentioned above, H_3_BTC (trimesic acid) and PTA (*p*-terephthalic acid) are also organic structures commonly used in the synthesis of MOFs. In addition, other features that could be evaluated in developing new gas sensors include multishelled materials. Multishelled copper oxides show improved sensor response for detecting ethanol when the number of shells in the copper oxides increases.^19^ Another study indicates that ZnO double-shell structures have increased sensitivity for detecting acetic acid compared to ZnO single-shell structures and nanoparticles.^20^ Therefore, developing new sensor materials with these features could be an effective strategy to enhance gas sensor properties, such as sensitivity and selectivity.

This work demonstrates the synthesis of NiO-yolk–shell (NiO-YS), NiO-yolk-double-shell (NiO-YDS), and NiO-yolk-triple-shell (NiO-YTS) sensors derived from Ni-BTC, NiO/Ni-BTC, and NiO/Ni-PTA composites, respectively, using the microwave-assisted solvothermal (MAS) method. The sensing properties of these materials were investigated against eight different VOCs, such as 2-butanone, 2-nonanone, 3-methyl-1-butanol, acetone, benzene, ethanol, methanol, and *m*-xylene. The NiO-YDS and NiO-YTS demonstrated interesting sensing properties for detecting *m*-xylene gas, showing excellent sensitivity and selectivity under dry conditions. Specifically, the NiO-YTS sensor from the NiO/Ni-PTA composite exhibited a sensitivity of 217.5% to *m*-xylene. In comparison, the NiO-YDS sensor from the NiO/Ni-BTC composite showed a sensitivity of 179.8% at 350 °C and in dry air. Therefore, these findings suggest that increasing the number of shells, that is, from a NiO double-shell (NiO-YDS) to a NiO triple-shell (NiO-YTS), improves the sensing performance, as previously described. The gas is likely trapped within the porous multishells of the materials, enhancing their sensitivity to the composites.

Because NiO-YTS stood out in preliminary experiments, this work explored its sensing characteristics more deeply, considering repeatability, stability, resistance, response under different concentrations, response in varying relative humidities (RH), and response–recovery times. The NiO-YTS-based sensor demonstrated stable and reproducible sensing performance for 100 ppm of *m*-xylene at 350 °C and in dry air, with a theoretical detection limit of 5.43 ppb and relatively fast response time (89 s) and recovery time (191 s). This study outlines a straightforward method for synthesizing NiO-YDS and NiO-YTS from NiO/Ni-BTC and NiO/PTA composites. It highlights its potential for improving the sensitivity and selectivity of *m*-xylene gas sensors. Therefore, these sensor materials demonstrated good performance in detecting *m*-xylene, which makes them suitable for various applications. For instance, they hold potential in fields such as breath analysis and prediagnosis, as xylene can be a biomarker for specific microorganisms.^21^ Furthermore, these sensors can assess indoor air quality in various industrial settings.

## Experimental Section

### Synthesis of NiO-YS via Ni-BTC

NiO-YS was produced from Ni-MOF (Ni-BTC) using the approach described by Kong et al.,^22^ with some modifications. Briefly, 872.40 mg (3.00 mmol) of Ni(NO_3_)_2_.6H_2_O (Sigma-Aldrich, >99.00%) and 210.10 mg (1.00 mmol) of trimesic acid (H_3_BTC) (Sigma-Aldrich, >99.00%) were dissolved in 60 mL of *N,N*-dimethylformamide (DMF). The solution was then put into a polytetrafluoroethylene (PTFE) autoclave with a stainless-steel seal for the MAS treatment (2.45 GHz/800 W)^23^ for 90 min at 150 °C. After the apparatus was cooled naturally to room temperature, the material was gathered using centrifugation. The precipitate was washed with DMF and ethanol three times and dried at 60 °C over 24 h. Green Ni-BTC was calcined at 500 °C for 120 min (5 °C min^–1^) in the air. The obtained black powder was named NiO-yolk–shell (NiO-YS).

### Synthesis of NiO-YTS from NiO/Ni-PTA Composite

The NiO-YTS sensor from the NiO/Ni-PTA composite was made utilizing the procedure described by Wang et al.,^24^ with some alterations. The preparation procedure of NiO-YTS was as follows: 74.69 mg (1.00 mmol) of NiO-YS (previously prepared) and 27.41 mg (0.165 mmol) of PTA (Sigma-Aldrich, >99.00%) were dissolved in a solution of 30.00 mL of DMF and 3.00 mL of deionized water. After being stirred for 30 min, the mixture was transferred to a PTFE autoclave with a stainless-steel seal for the MAS treatment at 150 °C for 90 min. The procedure for washing and drying the resulting solid was the same as for Ni-BTC. The obtained sample was a NiO/Ni-PTA composite, which showed a moss-green color and was represented as NiO-yolk-triple-shell (NiO-YTS).

### Synthesis of NiO-YDS from NiO/Ni-BTC Composite

**Figure 1:**
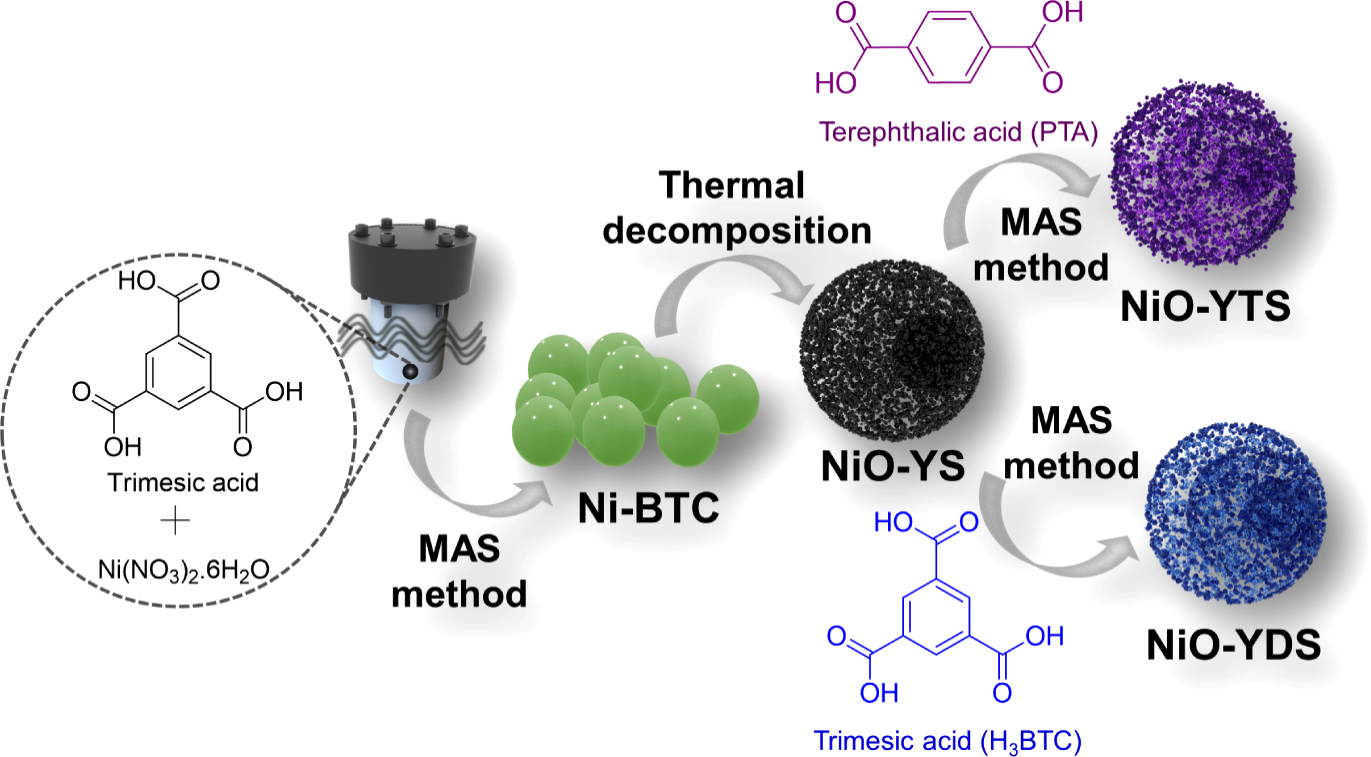
Design of the synthesis of Ni-BTC, NiO-YS, NiO-YDS, and NiO-YTS.

The NiO-YDS sensor from the NiO/Ni-BTC composite was synthesized using a similar procedure previously described to prepare NiO-YTS, which changed only the type of organic ligand. Briefly, 74.69 mg (1.00 mmol) of NiO and 34.67 mg (0.165 mmol) of H_3_BTC were dissolved in 3.00 mL of deionized water and 30.00 mL of DMF. After stirring for 30 min, the mixture was treated by the MAS method at 150 °C for 90 min. After the synthesis, the washing and drying procedures of the material were the same as for NiO/Ni-PTA. The obtained sample was a NiO/Ni-BTC composite, which showed a moss-green color and was denoted as NiO-yolk-double-shell (NiO-YDS). The synthesis procedure is shown in Figure 1.

### Characterization

The materials were characterized by thermogravimetric Analysis (TGA) (Q500 instrument in an air atmosphere extending from 50 to 600 °C, 5 °C min^–1^) and X-ray diffraction (XRD) analyses (Cu Kα radiation, λ = 1.5418 Å, operating at 10 mA, 30 kV) using a MiniFlex 300 diffractometer (Rigaku). Fourier transform infrared spectroscopy (FTIR) was carried out utilizing a PerkinElmer Spectrum Two spectrophotometer, operated in the 400–4000 cm^–1^ range. The surface area was established via the Brunauer–Emmett–Teller (BET) method, employing the Gemini VII model 2390t (Micromeritics). Field-emission scanning electron microscopy (FESEM) images and energy-dispersive X-ray (EDX) have been obtained at 2 kV by utilizing a JSM-6701F microscope (JEOL). The elemental analysis of carbon was performed in a PerkinElmer elemental analyzer (EA2400 CHN). For a complete analysis of the material’s nano/microstructures, transmission electron microscopy (TEM), high-resolution TEM (HRTEM), scanning transmission electron microscopy (STEM) using bright-field (BF) detectors, and selected area electron diffraction (SAED) have been carried out with a 200 kV JEM 2100F (JEOL) microscope. The X-ray photoelectron spectroscopy (XPS) and X-ray absorption spectroscopy (XAS) analyses have been performed according to previous work.^25^

### Gas Sensing Measurements

The procedures for sample preparation and VOC sensing measurements were like those in the previous study.^25,26^ All the measurements were carried out in synthetic air and at different temperatures (200 to 400 °C) under dry conditions. The subsequent math equations determined the sensor gas response (%):
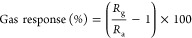
1

*R*_a_ and *R*_g_ are the electrical resistances of the sensor material in the air and the gas atmosphere, respectively. Furthermore, the response time is the period required to get 90% of the sensor’s stable value. Meanwhile, the recovery time represents the period for the resistance to decrease to 10% of its preliminary value in ambient air, starting when synthetic air was introduced to expel the test gas.^26,27^ The eight VOCs used in this study were 2-butanone (CAS 78-93-3), 2-nonanone (CAS 821-55-6), 3-methyl-1-butanol (CAS 123-51-3), acetone (CAS 67-64-1), benzene (CAS 71-43-2), ethanol (CAS 64-17-5), methanol (CAS 67-56-1), and *m*-xylene (CAS 108-38-3).

## Results and Discussion

**Figure 2:**
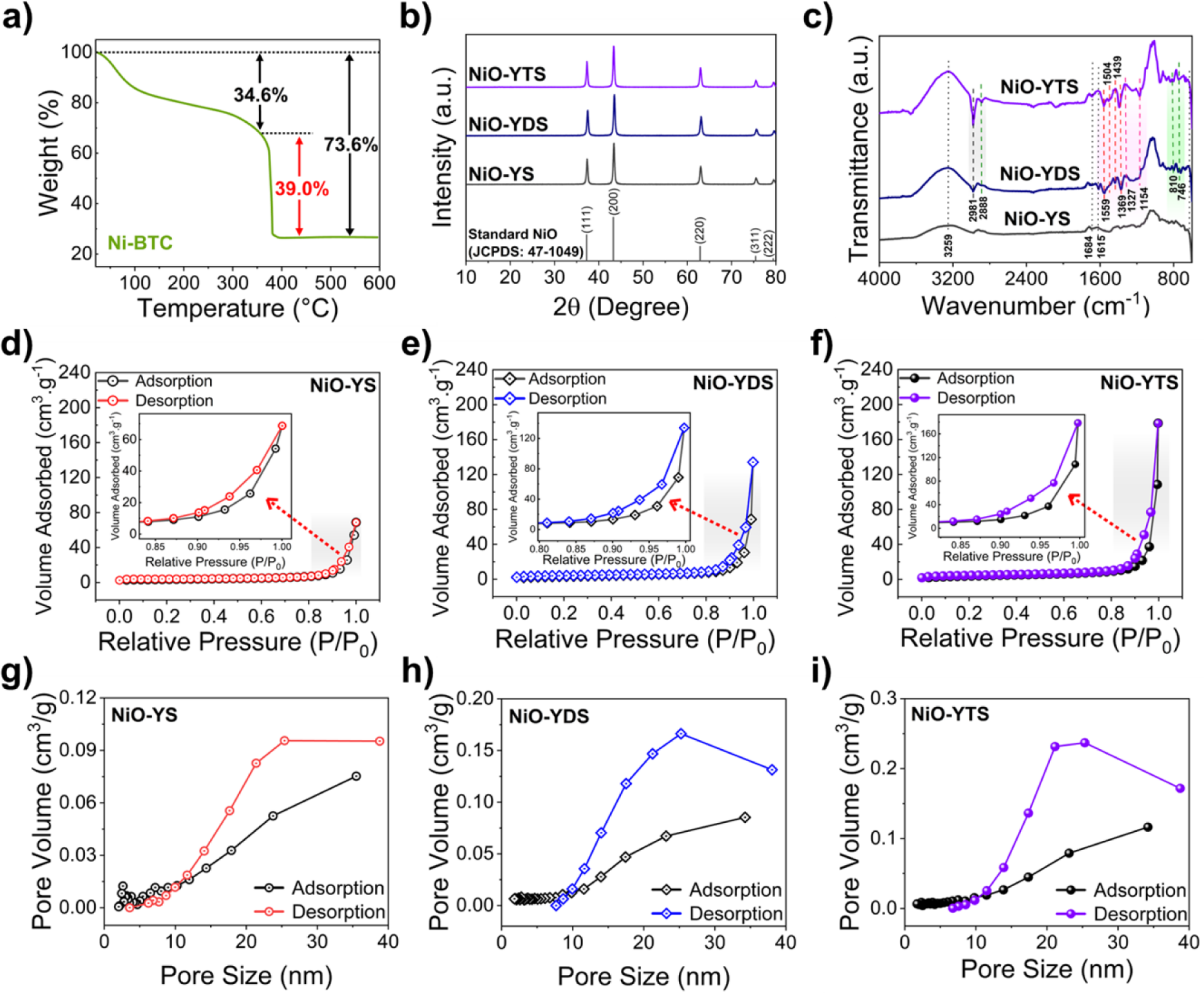
(a) TGA curve of Ni-BTC in air. (b) XRD patterns of synthesized NiO-YS, NiO-YDS, and NiO-YTS. (c) NiO-YS, NiO-YDS, and NiO-YTS FTIR spectra. N_2_ gas adsorption isotherms: (d) NiO-YS, (e) NiO-YDS, and (f) NiO-YTS. Pore size distributions during the adsorption and desorption processes of (g) NiO-YS, (h) NiO-YDS, and (i) NiO-YTS.

Thermogravimetric analysis (TGA) was conducted to assess the thermal decomposition of Ni-BTC to NiO-YS, as shown in Figure 2a. From this analysis, the thermal profile of Ni-BTC was evaluated, and the best temperature range for the calcination of Ni-BTC to NiO was selected. The initial weight loss of 34.6% occurs between 24 and 325 °C, corresponding to the evaporation of residual solvents and adsorbed gases such as O_2_, CO_2_, H_2_O, and DMF from Ni-BTC. When the temperature increased, complete thermal decomposition occurred, that is, the complete conversion of Ni-BTC to NiO-YS. This phase occurs between 370 and 400 °C, with a significant mass loss of 39.0%. At this stage, decomposition of the ligands of Ni-BTC results in a total weight loss of 73.6%. After this stage, there was no thermal process with the increased temperature, so NiO-YS could be formed at 400 °C. However, the temperature for calcining Ni-BTC was 500 °C, intending to reproduce the same morphology described in the literature.^22^ This temperature was sufficient to obtain Ni-BTC-derived crystalline NiO-YS.

X-ray diffraction (XRD) analysis was performed to determine NiO-YS, NiO-YDS, and NiO-YTS crystal structures, as illustrated in Figure 2b. The XRD measurements were carried out over a 2θ range of 10° to 80°. In this region, Figure 2, there are five diffraction peaks at 37.2, 43.3, 62.9, 75.4, and 79.4° in the XRD pattern of NiO-YS, which have been reported as (111), (200), (220), (311), and (222) crystalline lattices of NiO according to JCPDS No. 47-1049. The three materials had the same XRD pattern, representing that the two composites exhibit the same XRD pattern as the NiO-YS; that is, no additional diffraction peaks distinct from NiO-YS were seen in the NiO-YDS and NiO-YTS diffractograms. The thin organic layers in NiO-YDS and NiO-YTS are not detectable in XRD due to their low crystallinity and thickness, which mask their signal. Ni-BTC exhibited an XRD pattern like the one reported in the literature, as illustrated in Figure S1a.^22^

The Fourier transform infrared (FTIR) spectra of NiO-YS and NiO-YDS are presented in Figure 2c, covering the spectral range from 4000 to 600 cm^–1^. A broad band at 3259 cm^–1^ is responsible for (O–H) stretching vibrations in the NiO spectrum. This wide band is followed by the small bands at 1615–1684 cm^–1^, indicating the bending vibration mode in the H–O–H system. These results suggest the presence of H_2_O that has been physically adsorbed.^28^ The absorption bands below 1000 or 800 cm^–1^ are attributed to interatomic vibrations in SMO, such as NiO-YS.^29^ Therefore, the region between 672 and 600 cm^–1^ is due to Ni–O’s interatomic vibrations. The bands observed in the NiO-YS spectrum can be seen in the NiO-YDS and NiO-YTS spectra. In addition, NiO-YDS and NiO-YTS have similar FTIR bands to Ni-BTC, as displayed in Figure S1b. As shown in Figure 2c, there are two bands at 1559 and 1369 cm^–1^ in the NiO-YDS and NiO-YTS spectra, which are correlated by the symmetric and asymmetric stretching vibrations of the coordinated carboxyl (−COO^–^) group.^30^ The carbon–oxygen (C–O) bond stretching vibration in free carboxyl groups in NiO-YDS and NiO-YTS is responsible for the bands at 1327 and 1154 cm^–1^.

Moreover, the two small adsorption peaks at 1504 and 1439 cm^–1^ were attributed to the carbon–carbon (C–C) stretching vibration associated with the aromatic rings.^31^ Three peaks matched the *p*-aromatic C–H stretching bands at 2880, 810, and 746 cm^–1^.^24^ For NiO-YDS and NiO-YTS, the tiny absorption bands in the 692–648 cm^–1^ range indicate the torsional vibrations of the aromatic ring. The absorption band at 2981 cm^–1^ is a characteristic of the O–H hydrogen bond stretching vibrations for carboxylic acids, such as terephthalic acid and trimesic acid, but the signal does not appear in NiO-YS and Ni-BTC due to the establishment of the nickel–oxygen (Ni–O) bond in these materials.^32^ From this analysis, it is possible to identify the existence of a thin organic layer in NiO-YDS and NiO-YTS derived from NiO/Ni-BTC and NiO/Ni-PTA composites, which could impart different properties to the precursor material (NiO-YS), such as improving its sensor performance.

The nitrogen adsorption and desorption of the synthesized samples Ni-BTC, NiO-YS, NiO-YDS, and NiO-YTS are exhibited in Figures S1c and 2d–f, respectively. The isotherms exhibit a similar feature of a hysteresis loop around 0.8–1 of relative pressure (*P*/*P*_0_), which can be recognized as type IV based on the IUPAC classification.^33^ Also, Figures 2g–i present the pore size distributions during the adsorption and desorption processes of NiO-YS, NiO-YDS, and NiO-YTS, respectively. The structures exhibited pore widths ranging from 1 to 40 nm, with a higher concentration in the 10–20 nm range. These combined results indicate that the materials are mesoporous.

The pore size distribution and specific surface area of the materials are condensed in Table 1. As can be seen, NiO-YTS exhibited a higher and more satisfactory surface area, which is crucial for better diffusion of VOC molecules. Furthermore, the pore size of this material was lower than that of the other synthesized samples. This fact can be justified by adding a thin organic layer over NiO-YS, indicating a modification in the material properties and possibly increasing the sensor performance.

**Table 1:** Pore Size, Pore Volume, and BET Surface Area of the Synthesized Materials

Material	Pore size^b^ (nm)	Pore volume^a^ (cm^3^ g^–1^)	BET surface area (m^2^ g^–1^)
Ni-BTC	33.77	0.12	13.82
NiO-YS	27.96	0.08	11.47
NiO-YDS	33.98	0.11	12.47
NiO-YTS	16.54	0.06	13.96

a Single point adsorption total pore volume of pores less than 1952.438 Å diameter at *P*/*P*_0_ = 0.9900000.

b Adsorption average pore diameter (4 V/A by BET).

**Figure 3:**
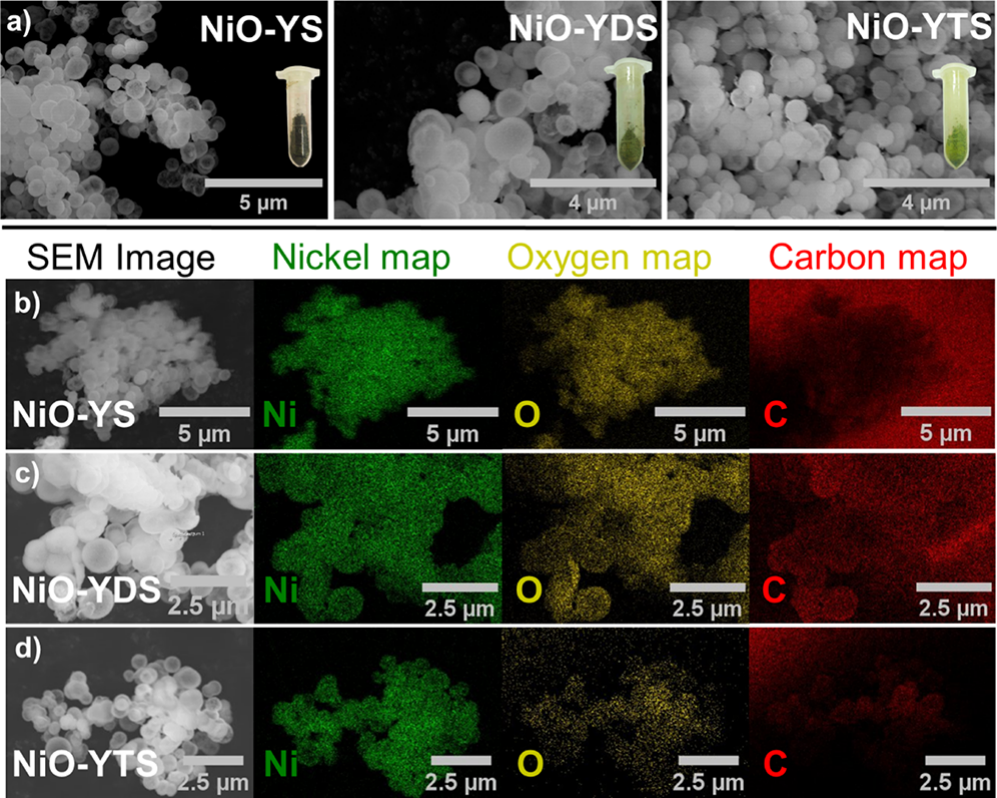
SEM images and EDX elemental maps: (a) SEM images of NiO-YS, NiO-YDS, and NiO-YTS. (b–d) EDX elemental maps of representative particles: (b) NiO-YS, (c) NiO-YDS, and (d) NiO-YTS. Elemental maps of Ni (green), O (yellow), and C (red).

The morphologies and EDX elemental maps of representative particles of Ni-BTC, NiO-YS, NiO-YDS, and NiO-YTS were characterized by FESEM, as represented in Figures S2 and 3, respectively. Ni-BTC displays a spherical morphology and green color, as seen in Figure S2, and its size was estimated to be 1 μm as established on the SEM images. After the calcination of the Ni-BTC precursor into NiO-YS, the morphology was the same as that found for Ni-BTC, and the color of the resulting material changed from green to black, as shown in Figure 3a. In addition, the sphere morphology was maintained with regular uniformity and smooth organization after NiO-YDS and NiO-YTS were synthesized from NiO/Ni-BTC and NiO/Ni-PTA composites, respectively, as indicated in Figure 3a. However, there are changes in the color of the NiO-YS material after synthesis; that is, the pure NiO-YS (black) becomes moss green when transformed into the NiO-YDS and NiO-YTS after the synthesis procedures, as shown in Figure 3a. Figures 3b–d (column 1) display SEM images of the region where the EDX maps were collected. The EDX elemental maps of nickel (Ni), oxygen (O), and carbon (C) in columns 2 to 4 illustrate how the different elements are distributed throughout the representative particles. As shown in Figures 3b–d, all materials are composed of Ni and O, but only NiO-YDS and NiO-YTS have C in their composition. Although carbon appears in the NiO-YS map, this does not mean this element is present in the sample. This signal comes from the carbon tape used in the analyses of the NiO-YDS and NiO-YTS elemental maps. Still, in addition to the tape signal, it is possible to observe the carbon in the specific region of the samples, showing that NiO-YDS and NiO-YTS presented the element carbon in their composition. This carbon could come from organic linkers (trimesic acid and terephthalic acid). Additionally, elemental analyses (CNH) were conducted to assess the carbon composition in the NiO-YDS and NiO-YTS samples, revealing approximately 2% carbon content in each. However, the NiO-YS sample exhibited 0% carbon content in its composition.

**Figure 4:**
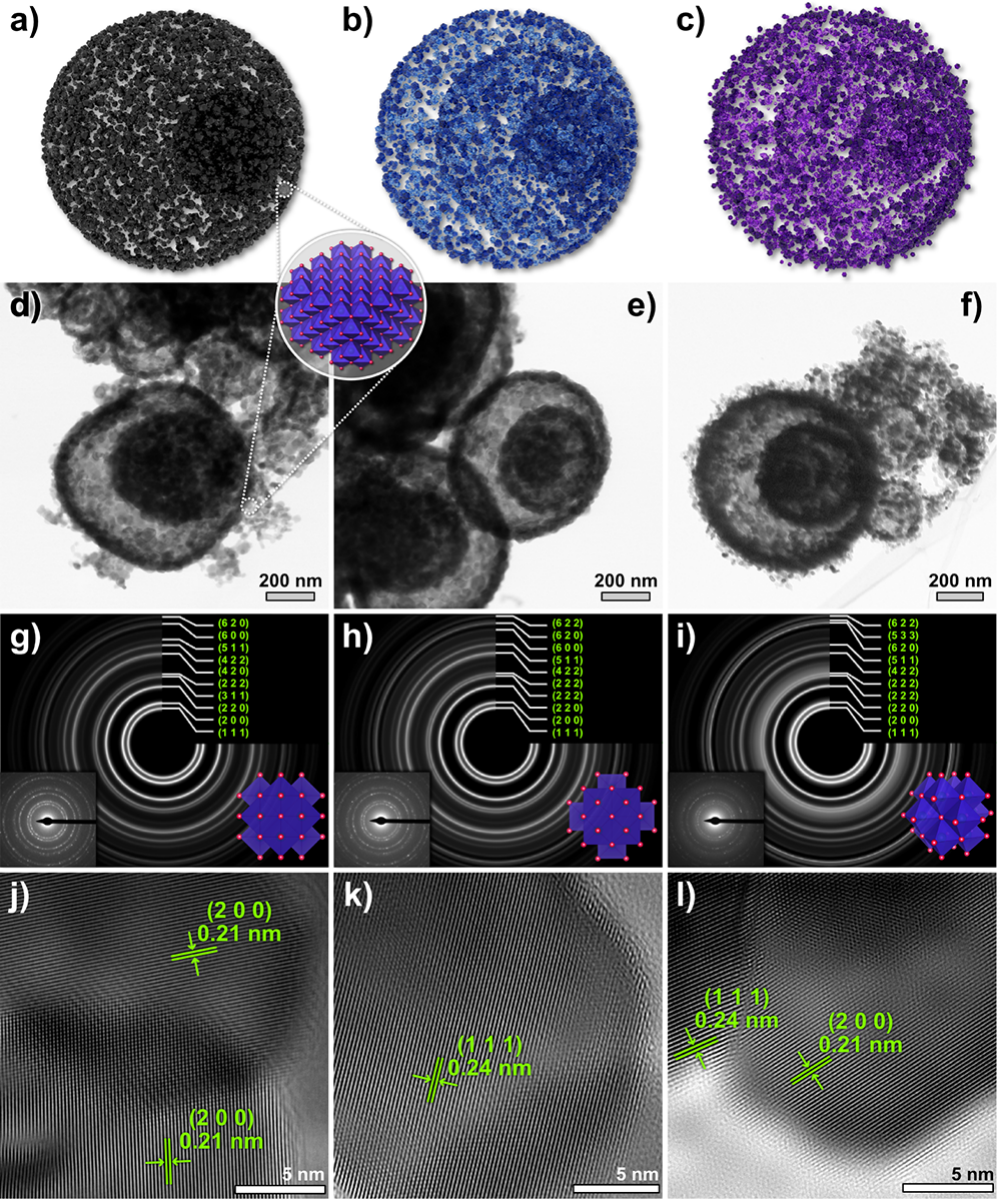
TEM characterization of NiO-based sensor materials. (a–c) 3D NiO, NiO-YDS, and NiO-YTS models represent core–shell, double-shell, and triple-shell structures, respectively. (d–f) STEM-BF images confirm the morphologies depicted in the models. (g–i) Rotational average diffraction patterns are indexed to the cubic NiO phase (COD 1010093) with insets showing the original patterns and accompanying cubic phase structure representations. (j–l) High-resolution TEM images of NiO, NiO-YDS, and NiO-YTS with indexed interplanar distances demonstrate the crystalline structures.

The TEM images in Figure 4 provide an in-depth analysis of the morphological and crystallographic properties of NiO-based structures. The 3D representations of NiO, NiO-YDS, and NiO-YTS, shown in Figures 4a–c, are confirmed by the STEM-BF images in Figures 4d–f. The NiO core–shell structure suggests a controlled synthesis route to maximize the surface area while maintaining a compact form. The additional shells in NiO-YDS and NiO-YTS leads to a progressive increase in surface complexity, facilitating enhanced gas interactions due to a higher surface area and potentially more active sites. The darker contrast at the core and lighter contrast at the shells in these images may also indicate the density and compositional variations across the different layers of the materials. With the selected area electron diffraction (SAED) patterns, rotational averaging was applied to index the cubic NiO phase from COD 1010093 (Figure 4g–i). Analyzing the rotational average, we can confirm that the degree of crystallinity is preserved across the samples. The insets show the original diffraction patterns, demonstrating uniform ring patterns without distortion. The structural representation of the cubic phase from different perspectives provides a visual illustration of the known crystal structure.

The HRTEM images exhibit lattice fringes, allowing for indexing of the interplanar planes (200) and (111), corresponding to distances of 0.21 and 0.24 nm, respectively, to the cubic phase of NiO, which confirms the structural integrity at the atomic level. The preservation of interplanar spacing in NiO-YDS and NiO-YTS, alongside the increasing shell complexity, suggests that the multishell synthesis process carefully controls the morphology without compromising the crystalline structure.

**Figure 5:**
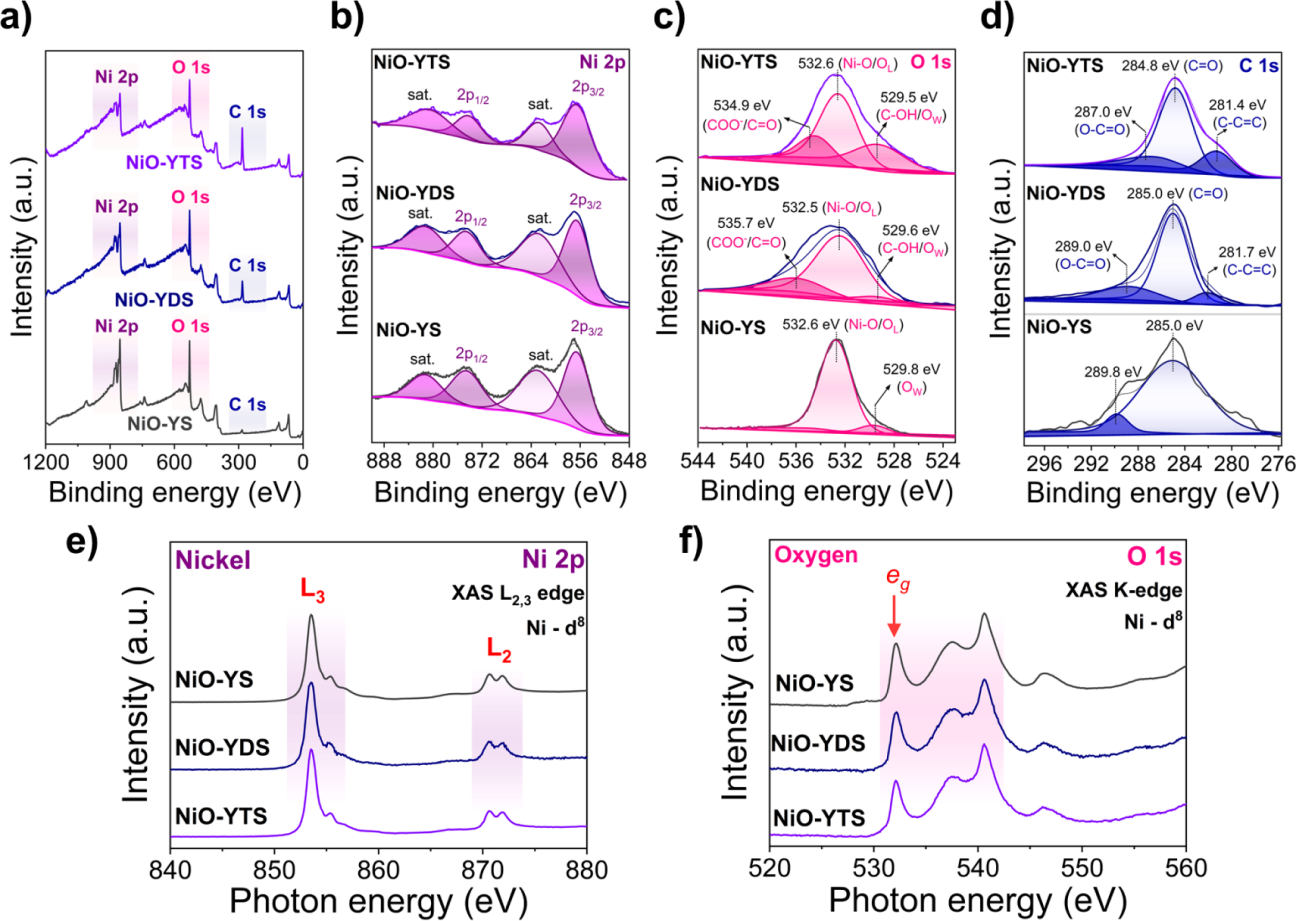
(a) XPS survey of NiO-YS, NiO-YDS, and NiO-YTS. High-resolution XPS spectra of (b) Ni 2p, (c) O 1s, and (d) C 1s for NiO-YS, NiO-YDS, and NiO-YTS. (e) Ni 2p L-edge XAS spectra and (f) O 1s K-edge XAS spectra of the 3d for NiO-YS, NiO-YDS, and NiO-YTS.

The X-ray photoelectron spectroscopy (XPS) and X-ray absorption spectroscopy (XAS) analyses have been performed to identify the chemical states, the local atomic structures, surface compositions, and electronic states of NiO-YS, NiO-YDS, and NiO-YTS, as displayed in Figure 5. As illustrated in Figure 5a, the presence of nickel (Ni), oxygen (O), and carbon (C) was observed in all three samples, which indicates the success in synthesizing NiO-YS, NiO-YDS, and NiO-YTS. High-resolution XPS (HRXPS) spectra of Ni 2p, O 1s, and C 1s for NiO-YS, NiO-YDS, and NiO-YTS are shown in Figures 5b–d, respectively. In Figure 5b, the HRXPS spectra of nickel in the three materials displayed deconvoluted peaks of Ni 2p at 856.7–857.1 and 874.5–874.8 eV, representing the spin orbits Ni 2p_3/2_ and Ni 2p_1/2_, respectively. Additionally, two peaks at 863.0–863.2 and 881.1–881.7 eV were related to the shakeup satellite (Sat.) peaks of Ni 2p_3/2_ and Ni 2p_1/2_, respectively. This finding confirms that the peaks are typical of Ni^2+^ species.^34,35^

Figure 5c presents the HRXPS spectra of O 1s for the three materials. It can be observed that, in the case of NiO-YS, two deconvoluted O 1s peaks appear at 532.6 and 529.8 eV. These peaks have been assigned to lattice oxygen (O_L_) and oxygen from surface species adsorbed, such as water (O_W_).^36^ The NiO-YDS exhibited three deconvoluted peaks at 529.6, 532.5, and 535.7 eV, attributed to oxygen in the carboxyl group (C–OH)/adsorbed water (O_W_), nickel–oxygen (Ni–O)/ lattice oxygen (O_L_), and carboxylate group (COO^–^/C=O), respectively.^37^ Similarly, the NiO-YTS displayed three deconvoluted peaks at 529.5, 532.6, and 534.9 eV, with a little difference in the binding energy and intensity values compared to NiO-YDS. These three deconvoluted peaks are associated with oxygen in the carboxyl group (C–OH)/adsorbed water (O_W_), nickel–oxygen (Ni–O)/ lattice oxygen (O_L_), and carboxylate group (COO^–^/C=O), respectively.^37,38^

Figure 5d illustrates that the C 1s spectrum is deconvoluted in three peaks positioned at 281.7, 285.0, and 289.0 eV for NiO-YDS and at 281.4, 284.8, and 287.0 eV for NiO-YTS. These peaks refer to the groups C–C=C, C=O, and O–C=O, respectively.^39^ Although the signal for C 1s is observed in NiO-YS, this is attributed to carbon from the tape used in the analysis. The XPS peak configurations in both NiO-YDS and NiO-YTS are similar, indicating the creation of a thin organic layer on NiO-YS through synthesis with organic linkers like trimesic acid and terephthalic acid to produce the NiO/Ni-BTC and NiO/Ni-PTA composites. The uniformity of peak patterns underscores XPS’s significance as a valuable surface analysis technique.

Figure 5e,f illustrates X-ray absorption spectroscopy (XAS) measurements of the nickel L_2,3_-edge and oxygen K-edge for NiO-YS, NiO-YDS, and NiO-YTS, all indicating consistent results. Additionally, the photon energies for the three samples displayed identical values for the XAS L_2,3_-edge in Ni 2p, as shown in Figure 5e. As previously detailed in a study on NiO microrods,^25^ the 2p core electron transition occurs through the excitation of electrons at the 2p level to an unoccupied state where the edge dominates the 3d empty states.^40^ Nickel atoms in NiO-YS are present in the Ni^2+^ configuration, which is a high-spin configuration.^41^ NiO-YS, NiO-YDS, and NiO-YTS share the same Ni^2+^ configuration, as these materials exhibit identical XAS spectra profiles. Figure 5f displays the O 1s spectra of NiO-YS, NiO-YDS, and NiO-YTS, revealing a consistent profile among the three samples. Interactions of oxygen species with 3d orbitals of nickel are responsible for the photon energy signals at 532.2 eV, representing, *e*_g_, hybridization. Additionally, hybridization between oxygen and Ni 4s and Ni 4p orbitals is represented by photon energy peaks at 537.5 and 540.6 eV, respectively.^41,42^

**Figure 6:**
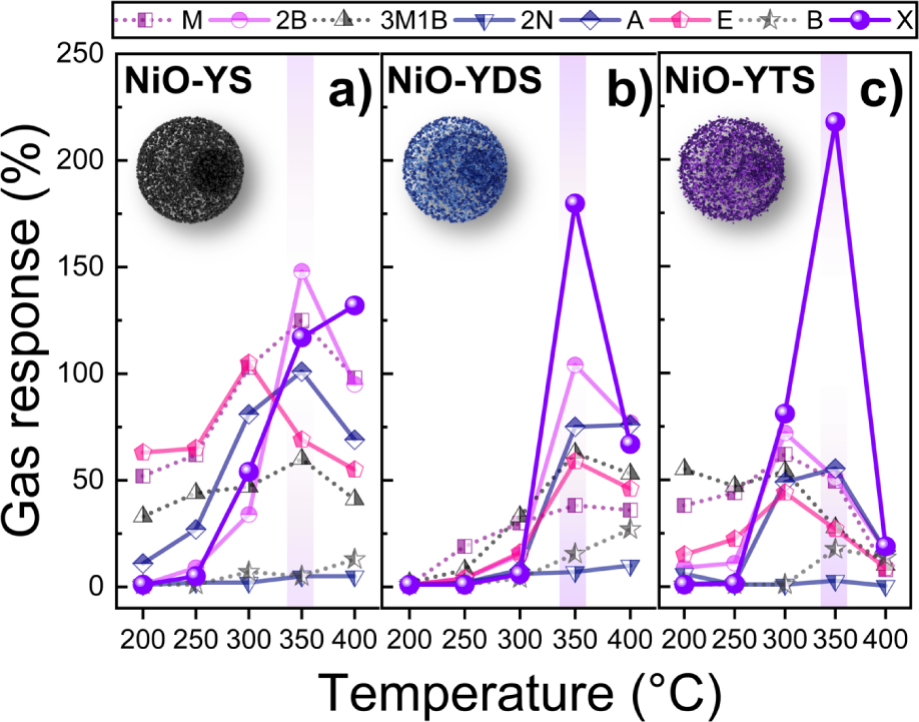
Sensing responses at 200 to 400 °C in 100 ppm of eight VOCs: methanol (M), 2-butanone (2B), 3-methyl-1-butanol (3M1B), 2-nonanone (2N), acetone (A), ethanol (E), benzene (B), and *m*-xylene (X). (a) NiO-YS, (b) NiO-YDS, and (c) NiO-YTS.

The gas detection performances of NiO-YS, NiO-YDS, and NiO-YTS at temperatures of 200 to 400 °C and dry air against eight VOCs are shown in Figure 6. As depicted in Figure 6a, the NiO-YS sensor detected six VOCs, such as 2-butanone (2B), 3-methyl-1-butanol (3M1B), ethanol (E), acetone (A), methanol (M), and *m*-xylene (X) at temperatures of 300, 350, and 400 °C, but this sensor did not exhibit a significant selectivity to any VOC. On the other hand, the NiO-derived sensors, that is, NiO-YDS and NiO-YTS, showed selective detection of *m*-xylene (X) at 350 °C (Figure 6b,c, respectively). As indicated in Figure 6b,c, the *m*-xylene responses were increased for these two sensors, while the responses for the other VOCs were decreased.

**Figure 7:**
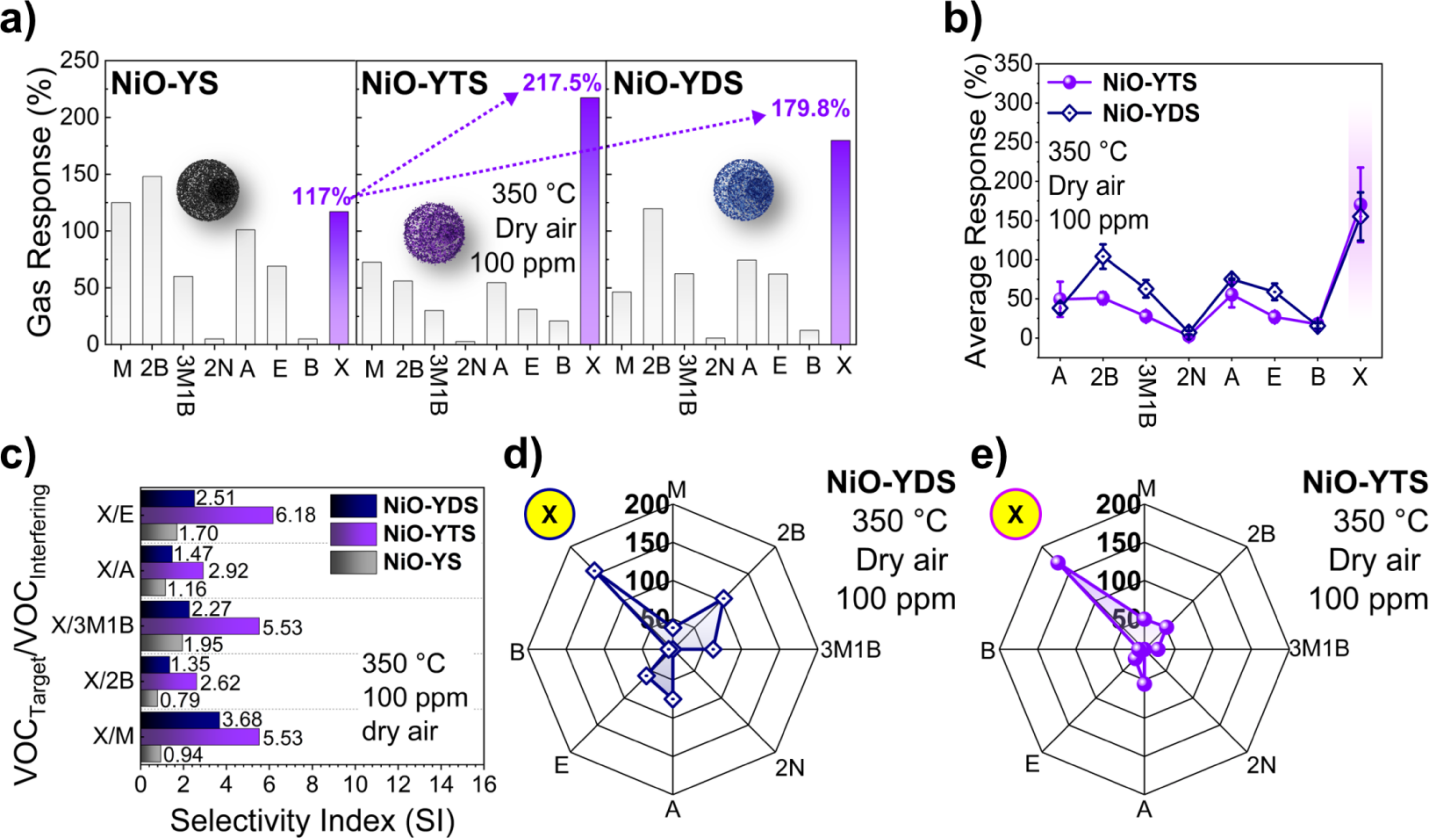
(a) Sensing responses of NiO-YS, NiO-YDS, and NiO-YTS at 350 °C to 100 ppm of eight VOCs: methanol (M), 2-butanone (2B), 3-methyl-1-butanol (3M1B), 2-nonanone (2N), acetone (A), ethanol (E), benzene (B), and *m*-xylene (X). (b) Average response of NiO-YDS and NiO-YTS at 350 °C in dry air. (c) Selectivity index (SI). (d,e) Selectivity radar graph.

Figure 7a shows the sensing responses of NiO-YS, NiO-YDS, and NiO-YTS at 350 °C to 100 ppm of the eight VOCs described in Figure 6. As indicated in Figure 7a, the gas detection performance of *m*-xylene to NiO-YDS (179.8%) and NiO-YTS (217.5%) increased when compared to that of NiO-YS (117%). On the other hand, the gas detection performance of other VOCs has decreased. Therefore, NiO-YDS and NiO-YTS showed a selectivity higher than that of NiO-YS in detecting *m*-xylene. To confirm the gas detection performance of the NiO-YDS and NiO-YTS sensors, we performed the measurements in triplicate under the same conditions (100 ppm, 350 °C, and dry air) against the eight VOCs to determine the average sensor response, as shown in Figure 7b. The NiO-YDS sensor showed an average response of 155.2 ± 30.8%, while the NiO-YTS sensor exhibited an average response of 170.1 ± 47.6%.

The selectivity index (SI) of the NiO, NiO-YDS, and NiO-YTS sensors was determined from the relationship between the responses of the target gas (*m*-xylene) and the interfering gases (methanol, 2-butanone, 3-methyl-1-butanol, acetone, and ethanol). The selectivity index (SI) is the fraction of a target gas response to an interfering gas response (*R*_VOC-target_/*R*_VOC-interfering_).^43^ As shown in Figure 7c, when comparing *m*-xylene with the other interfering VOCs, NiO-YTS was the material with the highest SI of all of the sensors in the study. For instance, the SI for NiO-YTS between *m*-xylene and methanol was 5.53 and 1.51 times greater than the SI for NiO-YDS (3.68) and 5.88 times greater than the SI for NiO-YS (0.94), while 2-butanone was the interfering VOC with the lowest SI value (2.62) for NiO-YTS compared to *m*-xylene. However, NiO-YTS has SI values 1.94 and 3.32 times higher than NiO-YDS and NiO-YS, respectively. For other interfering gases, the values of SI for NiO-YTS were 5.53, 2.92, and 6.18 for 3-methyl-1-butanol, acetone, and ethanol, respectively; these values are greater than those found for NiO-YS and NiO-YDS. Therefore, from these SI results, we can conclude that NiO-YDS and NiO-YTS exhibited sensing performances higher than those of NiO-YS at 350 °C in dry conditions with high selectivities for the detection of *m*-xylene, as shown in Figure 7d,e, respectively. However, when we compared the two NiO-derived sensors, the NiO-YTS material exhibited greater attributes, showing a higher sensitivity and SI to detect *m*-xylene. These results can be explained by the BET surface area of the sensors, where the NiO-YTS sensor (13.96 m^2^ g^–1^) has a higher BET surface area than the NiO-YS (11.47 m^2^ g^–1^) and NiO-YDS (12.47 m^2^ g^–1^) sensors, as shown in Table 1.

**Figure 8:**
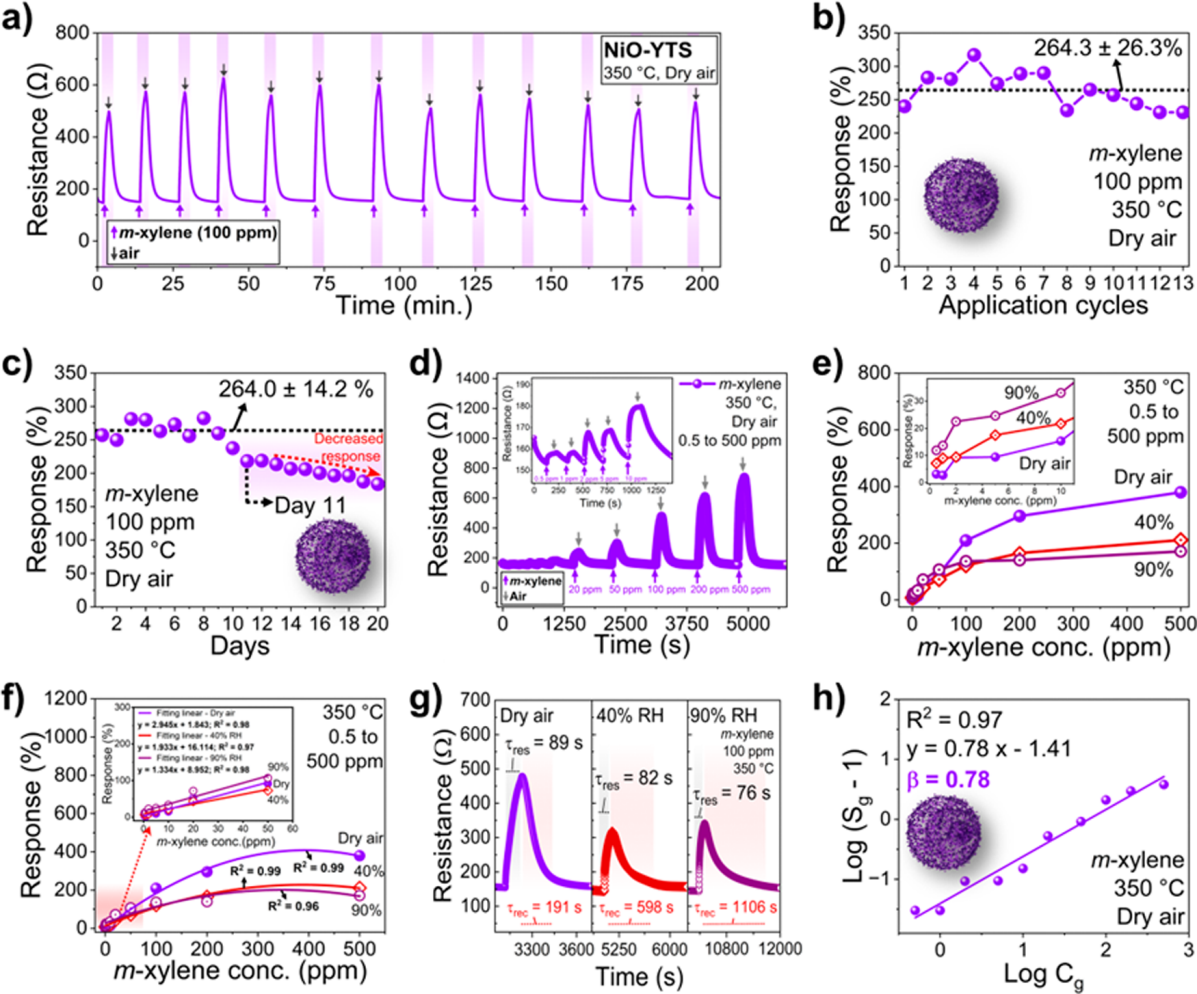
Sensing performance of the NiO-YTS sensor: (a) repeatability of the sensor to 100 ppm of *m*-xylene at 350 °C and in dry air; (b) gas response for *m*-xylene from repeatability analysis for the sensor; (c) sensor stability in 20 days; (d) dynamic sensing transients of the gas sensor based on NiO-YTS at 350 °C to 100 ppm of *m*-xylene under dry conditions; (e) gas response of the NiO-YTS sensor at 350 °C in different *m*-xylene concentrations (0.5 to 500 ppm) under different RH; (f) polynomial fit and linear fitting curves of response versus different *m*-xylene concentrations at different RH; (g) response time and recovery time curves for the NiO-YTS sensor to 100 ppm of *m*-xylene at 350 °C for different RH; (h) log (*S*_g_ – 1) in function of log *C*_g_ of the NiO-YTS sensor.

The sensing performance of the NiO-YTS sensor toward *m*-xylene is illustrated in Figure 8. Figure 8a,b shows the repeatability analysis of the sensor for 100 ppm *m*-xylene at 350 °C and in dry air. The NiO-YTS sensor exhibited stable and reproducible sensing performance to 100 ppm of *m*-xylene over 13 repeated tests with an average response of 264.3 ± 26.3% under these conditions after the 13 application cycles. Therefore, this analysis suggests that NiO-YTS shows good reproducible behavior for detecting *m*-xylene when evaluated sequentially, that is, on the same operating day. Figure 8c illustrates the stability of the NiO-YTS sensor in detecting 100 ppm of *m*-xylene under optimal operating conditions (350 °C and dry air) over 20 days. The NiO-YTS sensor demonstrates a stable and reproducible sensing performance in the initial 10 days with an average response rate of 264.0 ± 14.2%. However, beyond the 10th day, the sensor’s response consistently decreases, indicating that the NiO-YTS sensor is affected over time. Additionally, the material’s heating and cooling cycles at 350 °C for 10 days could cause sensor poisoning and a decline in its response, attributed to organic layer degradation from periodic heating.

Figure 8d displays the dynamic sensing transients of the gas sensor based on NiO-YTS at 350 °C under dry conditions when exposed to *m*-xylene concentrations from 0.5 to 500 ppm. As shown in Figure 8d, increasing the *m*-xylene concentration intensifies resistance variation, indicating a potential method for estimating the *m*-xylene gas concentration based on resistance variation value. From this analysis, it is evident that even at a concentration of 0.5 ppm, the resistance of the NiO-YTS material still undergoes a significant change (Δ*R* = 4.95 Ω), indicating that the sensor can detect *m*-xylene at low concentrations. Furthermore, from this analysis, we can conclude that the sensor demonstrates great response and recovery properties and remarkable reversibility within the range of 0.5 to 500 ppm of *m*-xylene. In addition, Figure S3a,b shows the dynamic sensing transients of the gas sensor based on NiO-YTS at 350 °C under different relative humidity (RH) conditions when exposed to various *m*-xylene concentrations (0.5–500 ppm). These results suggest that, in humid conditions ranging from 40 to 90% RH, the concentration of *m*-xylene can also be estimated based on the resistance values. Furthermore, the sensor can detect *m*-xylene at low concentrations, and NiO-YTS demonstrates excellent response–recovery properties, along with remarkable reversibility under different *m*-xylene concentrations in humid conditions.

The response values are further studied versus various *m*-xylene concentrations (ranging from 0.5 to 500 ppm) under different RH. As depicted in Figure 8e, the sensor’s response increases with the rising *m*-xylene concentration. However, the response decreases as relative humidity increases from 0 to 90% in the 100–500 ppm concentration range. These results are expected, as numerous studies have shown that increased humidity leads to decreased sensor performance. Additionally, environmental humidity can pose challenges for gas sensor development, primarily because it affects the resistance and sensing performance of the materials.^44−46^ Therefore, all studies should consider relative humidities when developing gas sensors. On the other hand, when *m*-xylene concentrations range between 0.5 and 10 ppm, the sensor exhibits a higher response in elevated humidity, attributed to water adsorbed on the material’s surface, aiding in better adsorption of low gas concentrations compared to higher concentrations (100 to 500 ppm). Additionally, the sensor responses for intermediate *m*-xylene concentrations (20 to 50 ppm) indicate almost identical values across a humidity range from 0 to 90%, which suggests that the NiO-YTS material can withstand varying humidity levels of *m*-xylene concentrations.

Figure 8f displays the response’s polynomial fit and fitting curves versus different *m*-xylene concentrations at varying relative humidity (RH) levels. The graph illustrates the response values corresponding to *m*-xylene concentrations at different RH, enabling the determination of polynomial functions describing the relationship between sensor responses (*y*) and various *m*-xylene concentrations (*x*). Through polynomial function fitting, a power law relationship is revealed as follows: *y* = 3.092 + 2.064*x* – 0.003*x*^2^ (dry air), *y* = 12.588 + 1.110*x* – 0.001*x*^2^ (40% RH), and *y* = 28.015 + 0.998*x* – 0.001*x*^2^ (90% RH). These equations exhibit *R*^2^ values of 0.99, 0.99, and 0.96, respectively, indicating strong correlations between response values and *m*-xylene concentrations.^47^ Additionally, linear fitting curves at low concentrations (ranging from 0.5 to 10 ppm) reveal a nearly straight-line variation in the inset of Figure 8f. The interdependence between the response values and different *m*-xylene concentrations is expressed by the equations: *y* = 2.945*x* + 1.843 (dry), *y* = 1.933*x* + 16.114 (40%), and *y* = 1.334*x* + 8.952 (90%), with corresponding *R*^2^ values of 0.98, 0.97, and 0.98, respectively. The least-squares method was employed to calculate the theoretical detection limit of the NiO/Ni-PTA sensor under varying RH conditions.^48^ The estimated theoretical detection limit of the sensor in dry air was 5.43 ppb, whereas at 40 and 90% RH, the theoretical detection limits were estimated to be 7.52 and 7.25 ppb, respectively.

The response (τ_res_) and recovery times (τ_rec_) for the NiO-YTS sensor were determined using 100 ppm of *m*-xylene at 350 °C and different relative humidities (dry air, 40, and 90%). As illustrated in Figure 8g, NiO-YTS exhibited relatively fast response times under various humidity conditions for detecting *m*-xylene, with τ_res_ of 89, 82, and 76 s in dry air and 40 and 90% RH, respectively. These results demonstrate that the response time decreased as the RH increased, indicating a faster detection rate. However, the improvement in response time was accompanied by a reduction in sensor sensitivity attributed to the reaction between adsorbed oxygen species (O^–^) and water, forming hydroxyl species, as described by the following reaction: H_2_O + O^–^ → 2OH^–^ + electron.^49^ On the other hand, the recovery time experienced a substantial boost with the rise in RH. The τ_rec_ values were 191, 598, and 1106 s in dry air and 40 and 90% RH, respectively. These findings demonstrate a 6-fold increase in recovery time when the sensor analysis takes place in high RH conditions, attributed to adsorbed water in the material interacting more strongly with *m*-xylene, compromising sensor performance and leading to decreased sensor response and longer recovery times.

The gas absorption model of semiconductors establishes the relationship between the gas response (*S*_g_) and its concentration (*C*_g_), which is defined by eq 2:^50^
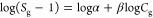
2

In brief, β represents the response order, indicating the types of oxygen species adsorbed on the material; for example, when β is approximately 0.5, the adsorbed species are O^2–^ and when β is close to 1, the adsorbed species are O^–^.^51^ Therefore, using eq 2, it is possible to estimate the oxygen species adsorbed in the NiO-YTS sensor. The relationship between log (*S*_g_ – 1) and log (*C*_g_) is illustrated in Figure 8h. As can be seen, there is a strong linear correlation (*R*^2^ = 0.97) between log (*S*_g_ – 1) and log (*C*_g_) for NiO-YTS at 350 °C under dry air conditions. From this, it was possible to determine the β value from the slope of the fitted line, which yielded a value of 0.78, suggesting that β is approximately equal to 1. Therefore, it is inferred that the dominant oxygen species adsorbed on the surface of the NiO-YTS material are O^–^.

Table 2 compares xylene sensing performance between material sensors based on various nanoparticles and core–shell and multishell SMO. The NiO-YTS sensor demonstrated superior performance in detecting xylene under varying humidity conditions compared with other material-based sensors discussed in the literature. Moreover, the NiO-YTS sensor exhibited faster response and recovery times than other sensors. This study, therefore, underscores the potential of the NiO-YTS sensor, highlighting its straightforward synthesis process and excellent xylene sensing capabilities. Furthermore, previous studies have shown that multishell materials have the potential to be applied as xylene sensors. These findings suggest that the NiO-YTS (triple-shell) sensor achieves better sensing performance than the NiO-YS (single-shell) and NiO-YDS (double-shell) sensors.

**Table 2:** Comparison of Xylene Sensing Performance of Different Material Sensors

Material	Temp. (°C)	Conc. (ppm)	Response (%)^a^	RH (%)	τ_res_/τ_rec_	ref.
CuCo_2_O_4_	150	200	75	-	241/750	(52)
α-MoO_3_	206	100	201	30	7/87	(53)
V_2_O_5_	290	100	175	30	21/134	(54)
Ag–Co_3_O_4_	250	50	147	Dry	1272/5989	(55)
Ag–Co_3_O_4_	250	50	125	40	-	(55)
Ni(OH)_2_/Co_3_O_4_	175	100	120	95	-	(56)
ZnCo_2_O_4_ HPA	260	200	46	40–50	1/12	(57)
ZnO/ZnCo_2_O_4_ core–shell	320	100	34.26^b^	-	-	(58)
Au@SnO_2_ core–shell	350	100	27.9^b^	-	-	(59)
NiCo_2_O_4_-double-shell	240	100	23.3^b^	Dry	15.4/30.5	(60)
NiO/NiWO_4_-yolk–shell	350	5	343.5^b^	-	-	(61)
NiO-yolk-double-shell	350	100	179.8	Dry	-	This work
NiO-yolk-triple-shell	350	100	217.5	Dry	89/191	This work
NiO-yolk-triple-shell	350	100	136.3	40	82/598	This work
NiO-yolk-triple-shell	350	100	121.6	90	76/1106	This work

a Determined from the gas response displayed in the works.

b Determined by *R*_a_/*R*_g_ or *R*_g_/*R*_a_.

**Figure 9:**
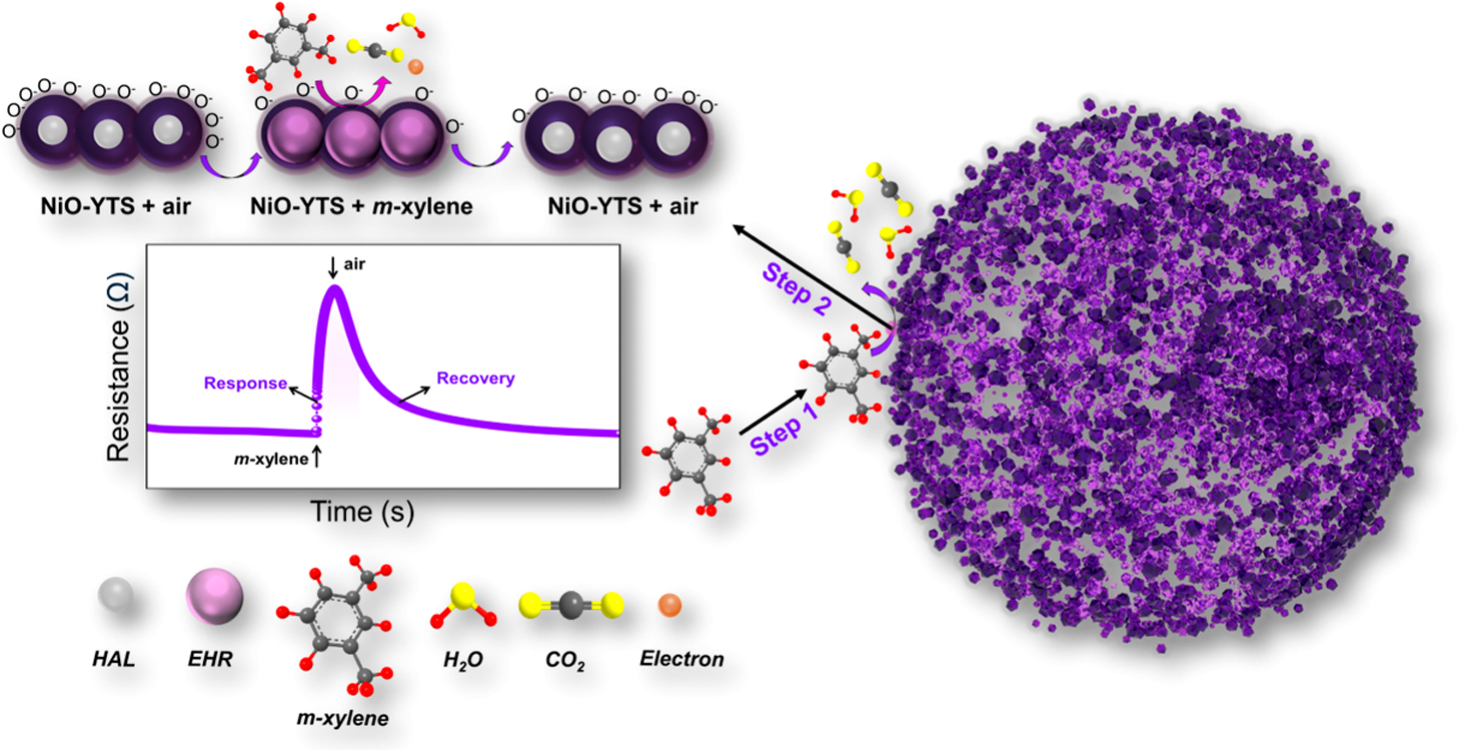
Schematic illustration of the *m*-xylene sensing mechanism for NiO-YTS.

Figure 9 shows the proposed sensing mechanism for detecting *m*-xylene by NiO-YTS. It is suggested that the sensing mechanism occurs in two steps, with the first step representing the capture of *m*-xylene by the organic layers in the NiO-YTS and the second step involving the oxidizing reaction between *m*-xylene and NiO. The *m*-xylene sensing mechanism for NiO-YTS is derived from SMO gas sensing, which relies on the resistance change of the material in response to various environmental gases.^62^ As shown in step 1, the gas *m*-xylene is captured by NiO-YTS with the assistance of organic layers (terephthalic acid linker) and traps the gas in its multiple shells of NiO, which could happen because *m*-xylene and organic layers interact through noncovalent interactions, such as π–π stacking interactions, which refer to interactions between aromatic systems containing π-bonds.^63,64^ Studies conducted by Guan et al. showed that carbon in MnWO4@C composite provides better adsorption sites and improves the xylene adsorption performance.^65^ After the organic layers and the multiple shells of NiO trap the gas in the NiO-YTS system, it is exposed to NiO, which acts as a sensor in step 2.

In *p*-type metal oxides like NiO, holes are the main charge carriers.^66^ As depicted in Figure 9, during step 2, exposure of the *p*-type NiO-YTS to air leads to the formation of a hole accumulation layer (HAL) on its surface due to the adsorption of ionized oxygen species. Consequently, an augmented concentration of holes enhances the material sensor’s conductivity and reduces its resistance.^67^ The operating temperature influences the configuration of adsorbed oxygen ions on the material surface and may include O_2_^–^, O^–^, and O^2–^ forms.^68^ From this, the NiO-YTS sensor exhibits chemisorbed oxygen ions on its surface, identified as O^–^ species at 350 °C, as previously defined by eq 2 and illustrated in Figure 8h. When the *p*-type NiO-YTS sensor is exposed to an *m*-xylene atmosphere, an oxidation reaction occurs between the *m*-xylene gas and the chemisorbed oxygen ions (O^–^), resulting in the production of carbon dioxide (CO_2_), water (H_2_O), and electrons (e^–^). Therefore, eq 3 describes this general reaction:
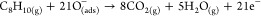
3

In addition, electron–hole recombination (EHR) occurs, indicating a reduction in hole concentrations in the HAL, which causes the thinning of the HAL and an increase in resistance when the material is exposed to *m*-xylene. After the response process, the NiO-YTS sensor chamber is cleaned with air. During this step, the sensor recovers, and its initial configuration with a thick HAL is re-established.

## Conclusions

NiO-YDS and NiO-YTS composites were synthesized selectively by using the MAS method to detect *m*-xylene under varying humidity conditions. These materials exhibited excellent sensitivity and selectivity for *m*-xylene detection under dry conditions, outperforming other evaluated VOCs. Furthermore, the NiO-YTS sensor (217.5%) demonstrated greater responsiveness and selectivity in detecting *m*-xylene than did the NiO-YDS sensor (179.8%). These findings suggest that increasing the number of shells, that is, from a NiO double-shell (NiO-YDS) to a NiO triple-shell (NiO-YTS), leads to improved sensing performance, as previously described. Notably, only the sensing performance of the NiO-YTS was assessed, including repeatability, stability, resistance, and response under different concentrations and humidity levels, as well as response–recovery times.

The NiO-YTS sensor showed remarkable stability, increased response with concentration, excellent performance under high humidity conditions, and reasonable response and recovery times. Therefore, this study underscores the promising characteristics of the NiO-YTS sensor, emphasizing its simple synthesis method and outstanding sensing performance in *m*-xylene detection. Additionally, it emphasizes the usefulness and capability of the NiO-YDS and NiO-YTS as prototypes for developing new material sensors with high selectivity for *m*-xylene, making them suitable for various application fields, such as breath analysis and prediagnosis, as xylene can serve as a biomarker for specific microorganisms. Furthermore, these sensors can assess indoor air quality in various industrial settings. Future studies could improve definite features such as sensitivity, stability, response and recovery times, and resistance to humid environments, utilizing different organic linkers and NiO-based Ni-MOFs with varied morphologies and multiple shells in synthesizing new composites.

## Supporting Information Available

XRD pattern of synthesized Ni-BTC; FTIR spectrum of Ni-BTC; N_2_ gas adsorption isotherms of Ni-BTC; SEM images of Ni-BTC; dynamic sensing transients of the gas sensor based on NiO/Ni-PTA at 350 °C to 100 ppm *m*-xylene under 40% RH and 90% RH (PDF)
