# Supplementary material for: Multiple-Yolk–Shell NiO Microspheres for Selective Detection of m-Xylene
Source: ACS Appl Mater Interfaces. 2024 Oct 5;16(41):55637–49. doi: 10.1021/acsami.4c09428 (PMC11492172; doi:10.1021/acsami.4c09428)
Supplement: Supplementary file 1 — am4c09428_si_001.pdf [file am4c09428_si_001.pdf]

Supporting Information

Multiple-Yolk-Shell NiO Microspheres for Selective Detection of m-Xylene

Reinaldo dos Santos Theodoro[a], Gustavo Sanghikian Marques dos Santos[a], Bruna Soares de Sá[a,b], Tarcísio Micheli Perfecto[c] and Diogo Paschoalini Volanti*[a]

[a]*Laboratory of Materials for Sustainability (LabMatSus), São Paulo State University (UNESP), Rua Cristóvão Colombo 2265, 15054-000 São José do Rio Preto, Brazil*

[b]*Brazilian Agricultural Research Corporation (EMBRAPA), São Carlos, SP 13560-970, Brazil*

[c]*Brazilian Center for Research in Energy and Materials (CNPEM), Campinas, SP, 13083-970, Brazil*

**Corresponding author. E-mail: diogo.volanti@unesp.br*

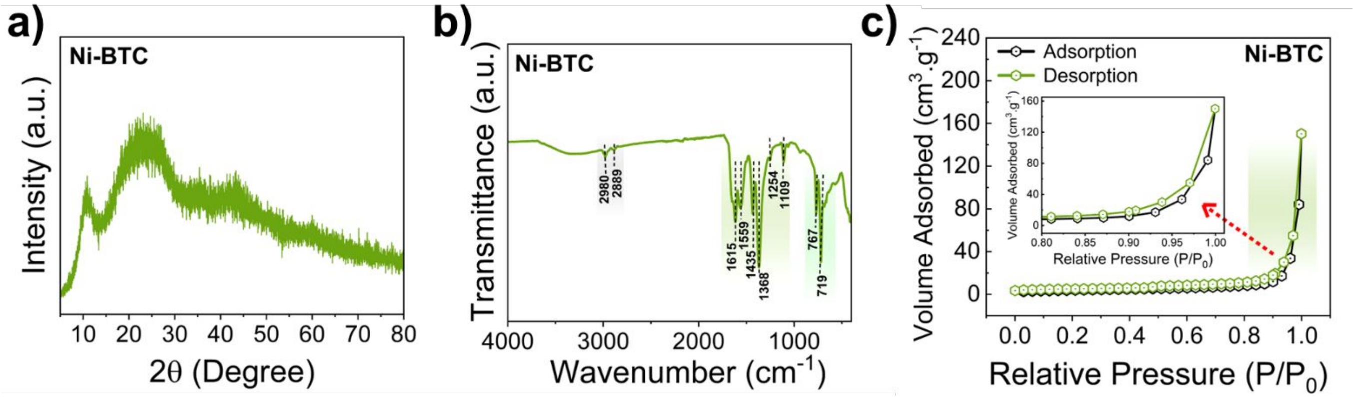

Figure S1. a) XRD pattern of synthesized Ni-BTC; **b)** FTIR spectrum of Ni-BTC; **c)** N_2 gas adsorption isotherms of Ni-BTC.

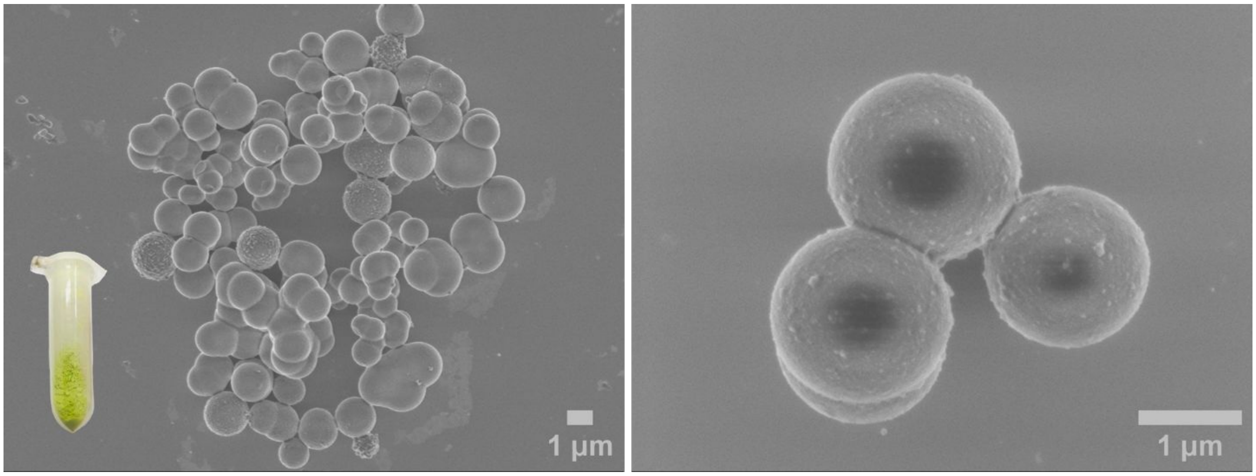

Figure S2. SEM images of Ni-BTC.

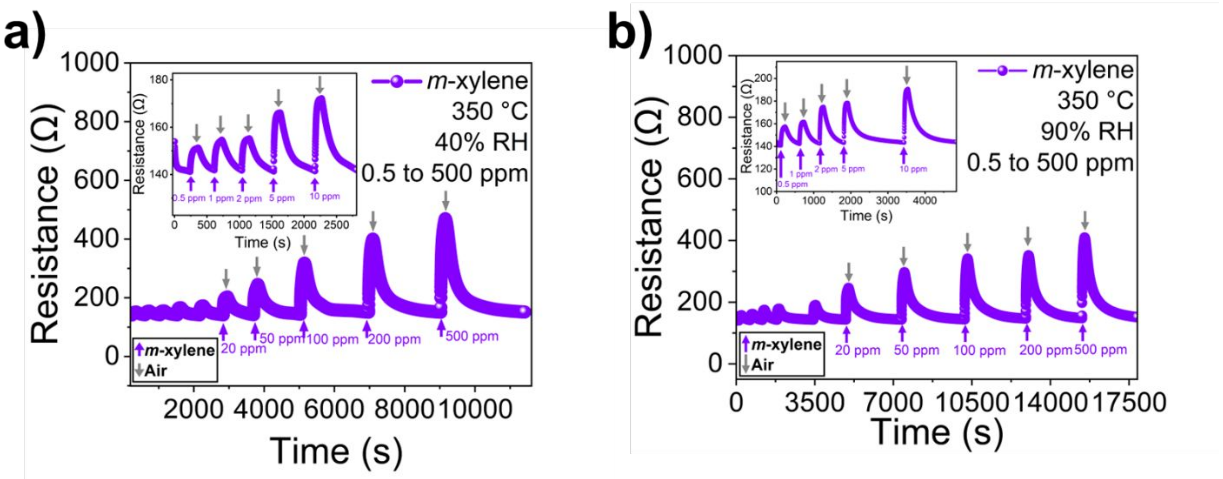

Figure S3. Dynamic sensing transients of the gas sensor based on NiO-YTS at 350 °C to 100 ppm *m*-xylene under: **a)** 40% RH and **b)** 90% RH.
